# Release of aluminium and thallium ions from uncoated food contact materials made of aluminium alloys into food and food simulant

**DOI:** 10.1371/journal.pone.0200778

**Published:** 2018-07-23

**Authors:** Stefan Sander, Oliver Kappenstein, Ingo Ebner, Kai-Andre Fritsch, Roman Schmidt, Karla Pfaff, Andreas Luch

**Affiliations:** German Federal Institute for Risk Assessment, Department of Chemical and Product Safety, National Reference Laboratory for Food Contact Materials, Berlin, Germany; University of South Carolina, UNITED STATES

## Abstract

In order to investigate the release of aluminium ions from food contact materials, three different types of uncoated aluminium menu trays for single use were tested with the foodstuffs sauerkraut juice, apple sauce and tomato puree, as well as with the food simulants 5 g/L citric acid solution and artificial tap water. To mimic a consumer relevant exposure scenario, the aluminium trays were studied using time and temperature gradients according to the Cook & Chill method, also taking into account storage time at elevated temperatures during the delivery period. The release of aluminium was found to exceed the specific release limit (SRL) of 5 mg aluminium per kilogram of food specified by the Council of Europe by up to six times. Furthermore, a release of thallium was also detected unexpectedly.

Kinetic studies showed a comparable behaviour in the release of aluminium, manganese and vanadium as components of the aluminium alloy itself. In contrast, thallium could be identified as a surface contaminant or impurity because of an entirely different kinetic curve. Kinetic studies also allowed activation energy calculations.

Additional camping saucepans were tested as an article for repeated use. In three subsequent release experiments with citric acid (5 g/L), artificial tap water and tomato puree as benchmark foodstuffs, the results were comparable to those of the uncoated wrought alloy aluminium trays.

## Introduction

Aluminium is critically discussed with regard to health aspects nowadays. It is ubiquitous and can be found in many foodstuffs which are the major source of human aluminium intake. Except for this exposure route, there are further sources of aluminium exposure for the general population, such as drinking water, drugs, cosmetics and industrial exposure. In 2008, the European Food Safety Authority (EFSA) issued an opinion on the safety of aluminium from dietary intake in which the typical aluminium content of unprocessed foodstuffs was reported at less than 5 mg per kg food, but it also referred to higher levels of 5 to 10 mg/kg. Based on animal studies, the EFSA derived a tolerable weekly intake of 1 mg aluminium per kg body weight per week. According to the EFSA assessment, the dietary intake of aluminium in the general population is between 0.2 to 1.5 mg per kilogram of body weight per week, equivalent to a daily intake of 1.7 to 13 mg of aluminium for a 60 kg adult [1]. The total aluminium content of foods comprises naturally present aluminium, aluminium from food additives and aluminium leaching into foods from food contact materials like aluminium foil, trays, cans, cookware, utensils and food packaging.

Aluminium is frequently used as a food contact material (FCM) e.g. in the form of foils or menu, baking and grill trays, as well as aluminium cans. It is sold for household and professional uses. Two different types of aluminium materials are distinguished: in unalloyed aluminium a minimum content of 99% aluminium must be met, whereas aluminium alloys can contain other elements such as silicon, iron, copper, manganese, magnesium, chromium, nickel, zinc, titanium and zirconium [2].

Thallium is a highly toxic metal which also occurs naturally in the environment and can therefore contaminate water and food. So far, no tolerable daily (or weekly) intake has been derived. In 2004, the German Federal Institute for Risk Assessment (BfR) recommended that the daily intake of thallium should not exceed 10 μg per person in the long term. The lowest observed adverse effect level (LOAEL) was reported to be 0.08 μg/kg per kg bodyweight [3].

Regulation (EC) No. 1935/2004 provides a harmonised legal framework for the safety of FCMs. Article 3 (1) states that FCMs must not release their constituents into food at levels that are harmful to human health, or change food composition or its organoleptic properties in an unacceptable way [4].

In the context of the Resolution of the Council of Europe CM/Res(2013)9 on metals and alloys used in food contact materials and articles, a technical guide for manufacturers and regulators was published by the European Directorate for the Quality of Medicines & Healthcare (EDQM) which defines specific release limits (SRL) for 21 metals [5]. The SRL is set at 5 mg aluminium /kg food and at 0.0001 mg thallium /kg food. An SRL for thallium of 0.0005 mg/kg was granted for a transitional period [6]. The technical guide proposes time and temperature conditions for release testing depending on the foreseeable uses [7]. Citric acid solution (5 g/L) and artificial tap water (ATW) are recommended for use as food simulants therein.

To check for compliance with the SRL, a risk-based control programme was conducted by the federal states of Germany in 2014. In the product group "articles for cooking, frying, baking and grilling made of aluminium", 29 samples were examined in line with the test conditions recommended by the Council of Europe and releases of aluminium with an average of 127 mg/kg were found in 19 samples [8]. A multi-factorial investigation by Fekete et al. revealed that temperature, contact time, pH and salt concentration are the main parameters influencing the release of aluminium [9]. High releases of aluminium into foodstuffs like tomato and sauerkraut, but also into 5 g/L citric acid from food contact materials made from aluminium are frequently reported and are regarded as not negligible by the authors [10–13].

The calculation of activation energy (*E*_*A*_*)* is a fundamental in chemistry and follows the Arrhenius equation, which is based on the time-temperature rule by Van’t Hoff [14–16]. Karbouj [17] did some fundamental research on the behaviour of the release of aluminium with calculations of its *E*_*A*_. She also showed the effect of passivation through pre-treatment [18].

In this work, a kinetic study was performed in order to understand the mechanism behind the release of aluminium by the simulant citric acid and to calculate the *E*_*A*_. For a comparison of the release of elements an aluminium tray, a grill plate and a foil were investigated.

Aluminium release in the more complex time and temperature conditions as applied in the Cook & Chill process was also investigated. The Cook & Chill process combines hot filling, a chilling period, storage at low temperatures and a re-heating up to 72°C core temperature (regeneration) [19]. Subsequently, meals prepared this way may be stored at elevated temperatures in the delivery area before being consumed. Uncoated aluminium trays are often used for this application. Mobile catering services often use the described procedure for the target group of particularly sensitive persons such as elderly people and young children [20]. Under the aspect of food hygiene, time and temperature conditions are specified in German standards DIN 10536 and DIN 10508 for the Cook & Chill and subsequent keep-warm processes [19, 21]. Our experiments were designed to mimic the time and temperature conditions specified for the Cook & Chill process combined with a storage period at elevated temperature (>65°C) in order to take the food delivery process into account. In addition to uncoated aluminium trays for single use, aluminium camping cookware for repeated use was tested in three subsequent release experiments. All samples were tested using the simulants citric acid solution (5 g/L) and ATW as proposed in the technical guide of the Council of Europe [5, 7]. Sauerkraut juice, apple sauce and tomato puree were used as benchmark foodstuffs.

## Experimental

### Investigated samples and benchmark foods

Aluminium foil and uncoated grill trays were purchased from retailers in Berlin. Aluminium camping saucepans and four different types of uncoated aluminium menu trays were purchased from German retailers through the internet. Trays consisted of either one, two or three compartments. Chamber volumes, contact areas and food simulants or foods used are listed in S1 Table.

The benchmark foods were sauerkraut juice (pH 3.7), apple sauce (diluted 1:1 (m/m) with ultrapure water, pH 3.4) and tomato puree (pH 3.5). The salt content of tomato puree and sauerkraut juice was labelled as 0.9% each. Food simulants were 5 g/L citric acid solution and artificial tap water prepared according to DIN 10531 [22].

### Kinetic studies on elemental release

Pieces of 5 x 10 cm in size were cut from the one-compartment aluminium menu trays, aluminium foil or grill trays respectively and submerged in 500 mL of citric acid (5 g/L) for 2 hours in an Erlenmeyer flask on a hotplate with temperature control (IKA RCT basic, ETS-D5). Aluminium release was investigated at 60, 70, 80, 90 and 100°C and temperatures were recorded with a temperature data logger (iButton, DS1922T). Each experiment was carried out in triplicate. At temperatures of 100°C and 90°C samples were taken every five minutes during the first 30 minutes, every ten minutes from 30 to 60 minutes and every 15 minutes thereafter. Sampling was carried out at 80, 70 and 60°C every 15 minutes in the first hour and every 20 minutes in the second hour. A peristaltic pump (ismatec, ISM941) which constantly pumped 10 mL/min in circulation and into a sample tube after switching a valve was used for sampling.

In addition, cooling down experiments were performed in which the aforementioned equipment was used. After assembling the equipment and installation of the aluminium foil piece, 500 mL of boiling 5 g/L citric acid solution was poured into the beaker which was immediately covered with a silicone sheet. The solution was allowed to cool down under constant stirring. Sampling was carried out every 5 minutes in the first 30 minutes and every 10 minutes from 30 to 120 minutes using a peristaltic pump. Samples of grill plates and aluminium foil were treated in the same way.

### Release experiments with uncoated aluminium trays under the conditions of the Cook & Chill process

Release experiments were carried out adhering to the conditions specified in DIN 10536 for the Cook & Chill method and DIN 10508 for temperature requirements for foodstuffs [19, 21]. Trays were filled with boiling liquid or food, covered and cooled down to 3°C within 90 minutes followed by storage at 3°C for three days. Finally, the aluminium trays were re-heated to a core temperature of 72°C for two minutes. To mimic mobile catering services, the trays were kept above 65°C for two hours. An analysis was made at four different points in time: 1^st^ after hot fill and cooling, 2^nd^ after storage at 3°C, 3^rd^ after re-heating to 72°C core temperature and 4^th^ after storage above 65°C for 2 hours. A blank sample of foodstuff or food simulant treated in the same way in a polypropylene beaker was also analysed at each time.

Three different types of tray were used for the investigations on aluminium trays. The one-compartment tray was tested with apple sauce and citric acid solution (5 g/L). The two-compartment trays were tested with apple sauce (small chamber) and sauerkraut juice (big chamber), citric acid solution (big chamber) and ATW (small chamber). In the three-compartment tray tomato puree was used for the biggest chamber, sauerkraut juice for the middle and apple sauce for the smallest chamber. With this tray, the simulants citric acid solution and ATW were tested separately by filling all chambers with each simulant. All trays were covered with an aluminium lid after hot filling. All tests were performed in duplicate.

### Release experiments in aluminium camping saucepans

Release studies were carried out as specified for repeated use articles in the technical guide for metals and alloys used in food contact materials and articles [7]. One litre of tomato puree, 5 g/L citric acid or artificial tap water were kept at boiling point in saucepans on a hot plate. Each camping saucepan underwent three subsequent release experiments. Two samples of each saucepan were taken after 30 and 60 minutes.

### Reagents and materials

Detailed information on chemicals and elemental standards including CAS numbers, purity or concentrations and supplier information can be found in S2 Table. Nitric acid was purified in a duoPUR quartz sub-boiling point distillation apparatus (MLS GmbH, Leutkirch, Germany). HPLC grade water (18.2 MΩ∙cm output quality) was obtained from Milli-Q water purification equipment (Merck Millipore, Darmstadt, Germany).

### Sample preparation of food simulants and benchmark foods

After release testing, two samples of food simulants of each chamber were diluted 1:10 with 3.5% HNO_3_ (containing 200 μg/L gold ions) and 150 μL of internal standard solution (ruthenium ions, 5 mg/L) were added to 15 mL. Food samples underwent microwave digestion in a single reaction chamber system (MLS, ultraclave II). For this, one gramme of homogenised sample, 500 μL of internal standard solution (ruthenium ions, 5 mg/l), 3 mL of water and 5 mL of concentrated nitric acid (69%) were mixed in the teflon extraction vessel of the microwave device. The final sample digestion step was carried out at 200°C for 26 minutes, whereby the maximum pressure amounted to 160 bar. Digested samples were diluted to a final volume of 50 mL with 3.5% HNO_3_ (containing 200 μg/L gold ions).

### Determination of elements by ICP-MS

Elements up to a weight of 117 u were measured with the collision cell technology as kinetic energy discrimination (KED) and a mixed gas of helium with 2% hydrogen on an ICP-MS system (Thermo Fisher Scientific, iCapQ). Elements heavier than 117 u were measured in standard mode. The elements, their SRLs and the isotopes which were chosen for measurement and their corresponding internal standards are shown in S3 Table. The system was operated with 1550 W of RF-power. The gas flow of the nebuliser, auxiliary gas and cooling gas were set to 1.1 L/min, 0.7 L/min and 14 L/min, respectively. Samples were measured with a dwell time of 0.01 s with 100 sweeps per reading. To account for dilution inaccuracies during sample preparation, the internal ruthenium standard was used to calculate a correction factor. A mixed internal standard solution containing rhodium and bismuth in 10% isopropanol and 3.5% nitrous acid was used as the injection standard with a concentration of 5 μg/L. The latter was added immediately before the solution was nebulised via the autosampler system (Elemental Service & Instruments GmbH, prepFAST). The same system was used to dilute samples exceeding the working range concentration by 60% of the highest value of the calibration curve, which was prepared to cover 0.001 to 0.5 times the respective SRL. Methods were validated and limits of detection (LOD) or limits of quantification (LOQ) were calculated according to DIN 32645 with measurements of blanks and processed blanks [23]. Validation data with LOD, LOQ and recovery at concentrations of 0.08, 0.8 and 8 times the SRL can be found in S4 Table. Data were processed with the Qtegra software (Thermo Fisher Scientific) and Excel (Microsoft).

## Results and discussion

### Investigation in the release kinetics

**Fig 1:**
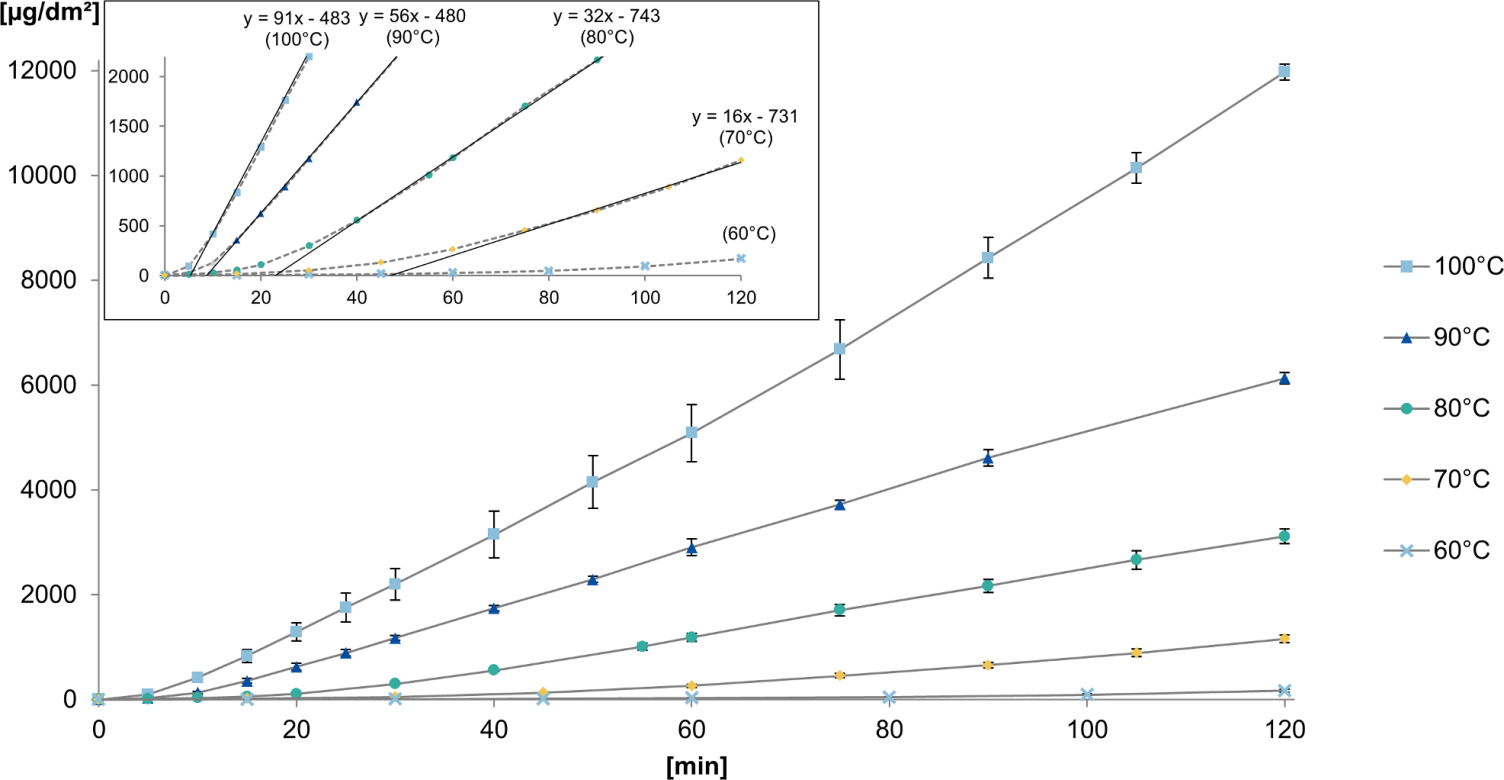
Kinetic behaviour of the release of aluminium in 5 g/L citric acid solution at temperatures of 60–100°C.

For the kinetic test, a piece with a 1 dm^2^ surface of a one-part aluminium tray was cut out and totally immersed in 5 g/L citric acid solution. The time-dependent aluminium release resembles the behaviour observed in lag time experiments with diffusion through barriers [24]. After an initial delay and a slow aluminium release, a linear release was observed as shown in Fig 1. This indicates that the release is determined by two different processes. A linear increase in dissolution of the plain metal was achieved after the slow initial dissolution of the oxide layer. The asymptote of that part of the function is used to determine the lag times of each experiment as shown in the inset to Fig 1. As linear behaviour could not be found at 60°C, the lag time could not be calculated due to the experimental observation time of 120 minutes being too short for reaching an equilibrium. With the lag times, the thickness of the oxide layer is calculated based on the released amount of aluminium from the known surface area, assuming a uniform layer and chemical composition of Al_2_O_3_ with a density of 3.94 g/cm^3^. The oxide layers were found to be 4–11 nm thick, which complies well with the literature [25]. Karbouj et al. showed the decrease in the release of aluminium following a pre-treatment in hot water near boiling point for a period of five hours. The longer the pre-treatment, the lower the release of aluminium in the following experiment in citric acid solution [18]. These findings underline the speed-reducing contribution of the oxide layer onto aluminium release as shown by the lag times.

With those kinetic results the activation energy (*E*_*A*_*)* can be calculated according to the Arrhenius equation [15, 16] by changing the equation as follows:

$$k=A∙e^{\frac{-E_{A}}{R∙T}}$$

$${-E}_{A}=R∙\frac{\ln\;\left(\frac{k_{2}}{k_{1}}\right)}{\frac{1}{T_{1}}-\frac{1}{T_{2}}}$$

$$E_{A}=-R∙\left[\frac{\Delta \ln\;k}{\Delta (\frac{1}{T})}\right]$$

where *k* = reaction rate constant, *A* = pre-expotential factor, *R* = universal gas constant and *T* = absolute temperature.

**Fig 2:**
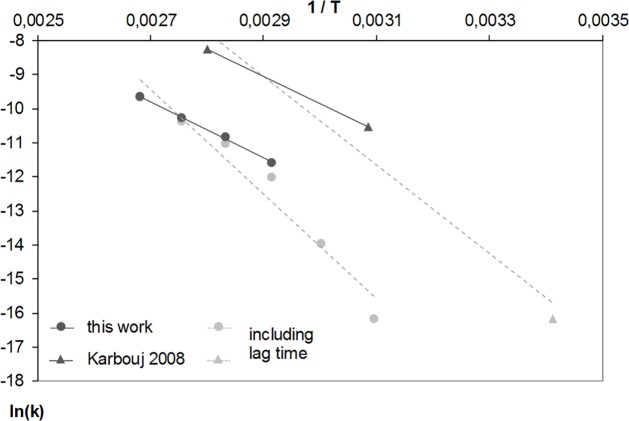
Arrhenius plot of aluminium releases for the temperature range 50 to 100°C compared to Karbouj’s data (dark grey: Data from constant release conditions, in grey: Data including lag time).

The activation energy can be derived from the slope of the Arrhenius plot to 62 kJ/mol. The effect of neglecting the influence of the initial oxide dissolution can be demonstrated by inserting the aluminium release values obtained after the complete 2-hour experiments for each temperature. As shown in Fig 2, this leads to a non-linear curve in the Arrhenius plot (grey circles). Fitted to a linear curve (grey line) this would result in a value of the activation energy of 128 kJ/mol. Comparable behaviour could be seen in the doctoral thesis of Karbouj [17]. For comparison, the reaction rates from Karbouj’s work were converted to mmol/dm^2^/s applying the details of that study i.e. 0.75 dm^2^ sample surface and 275 mL. Lag time and steady state linear releases are shown and taken into account for 84 and 51°C. Unnoted by the author, the equilibrium for 20°C was not reached in the experiments. The reported *E*_*A*_ of 110 kJ/mol should therefore be corrected. Considering only the values measured at temperatures of 84 and 51°C in the calculation, an *E*_*A*_ of 65 kJ/mol can be recalculated, confirming our findings.

**Fig 3:**
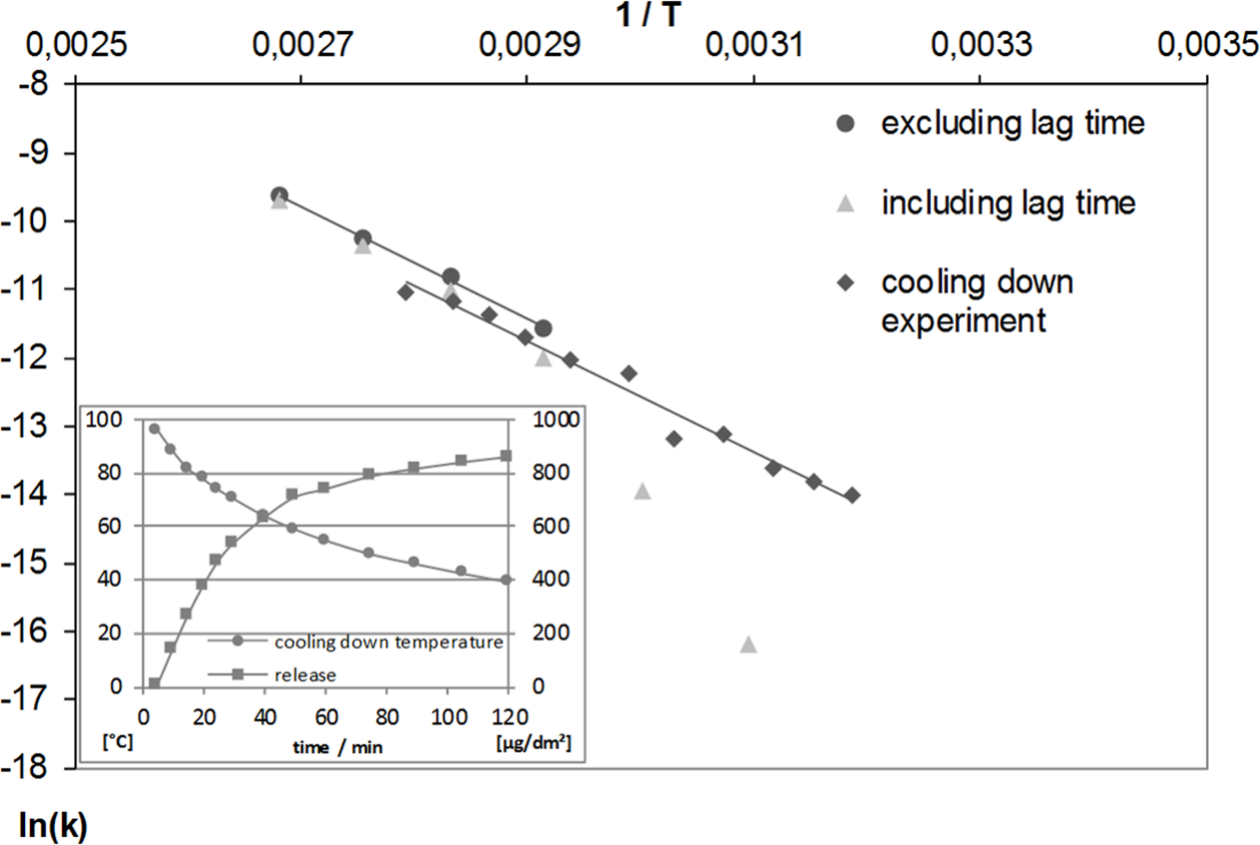
Arrhenius plot of aluminium releases for kinetic results from cooling down experiments between 90 and 40°C compared to the isothermal experiments (Inset: Course of temperature and aluminium release).

Release experiments for calculating the *E*_*A*_ are laborious and time consuming, especially if data points at lower temperatures are to be included. A new approach was introduced to overcome these difficulties and simplify the experimental work. Instead of conducting consecutive isothermal experiments, all necessary data were obtained in one single experiment. For this purpose, the release experiment was conducted by pouring boiling 5 g/L citric acid solution over the 1 dm^2^ aluminium piece and allowing the solution to cool down over a period of two hours. Samples were drawn every 5 minutes during the first 30 minutes and every 10 minutes thereafter while constantly monitoring the temperature. The temperature average of each sampling period was used for calculation when constructing the Arrhenius plot in Fig 3. The *E*_*A*_ was calculated as in the previous experiments. To enable comparison, the data of the previous experiments are shown together with the data from the cooling-down experiment. The calculated value for the *E*_*A*_ is 68 kJ/mol, which again matches up well with the previous findings.

**Fig 4:**
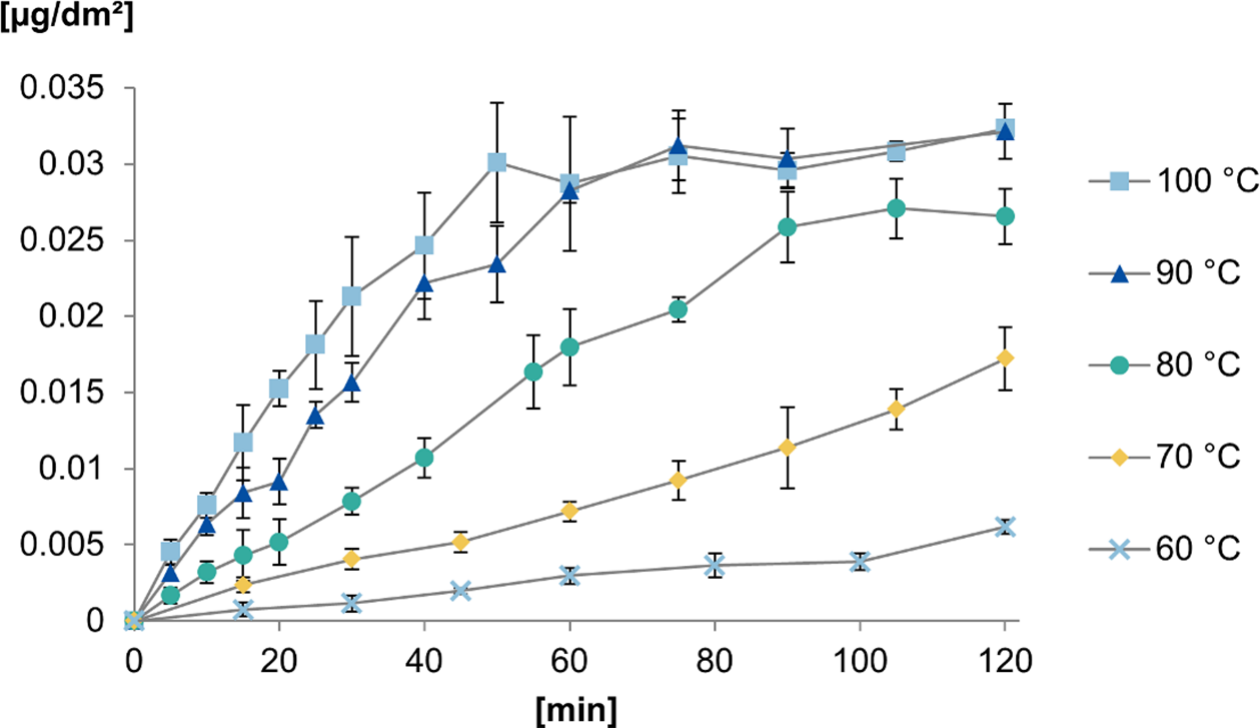
Release kinetics of thallium into citric acid solution (5 g/L) at temperatures of 60–100°C in increments of 10°C (n = 3).

In contrast to the release of aluminium, the release of thallium from the aluminium tray did not show any lag time as shown in Fig 4. Instead, the concentration of thallium increased nearly linearly up to a constant level. For 100°C and 90°C, the constant level was achieved after 60 minutes. At 80°C the release was slower but reached nearly the same level after 90 minutes. At temperatures of 70°C and 60°C, stagnation was not reached after two hours, but a significant release of thallium with a linear behaviour was also measured.

**Fig 5:**
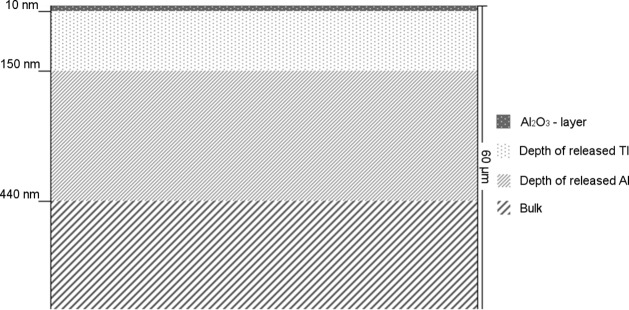
Distribution of Al_2_O_3_ and released thallium and aluminium after two hours at 100°C in 5 g/L citric acid solution.

Thallium release reaches its maximum of about 0.03 μg/dm^2^ after 50 minutes at 100°C. The absence of a lag time indicates that the release is independent of the oxide layer and that it is distributed homogeneously on the surface. The total release of almost 12 mg aluminium per dm^2^ after two hours corresponds to the removal of a layer of about 440 nm as shown in Fig 5. From the findings that after 50 minutes at 100°C the increase in the concentration of thallium almost ceased and 4.15 mg aluminium had been released by then, the thickness of the aluminium layer containing thallium can be calculated to be approximately 150 nm by using the volumetric formula of a cuboid with the surface of 1 dm^2^ and an unknown height. With an oxide layer of about 5–10 nm, thallium is distributed much deeper in the material than Al_2_O_3_ but it still seems to be near the surface.

**Fig 6:**
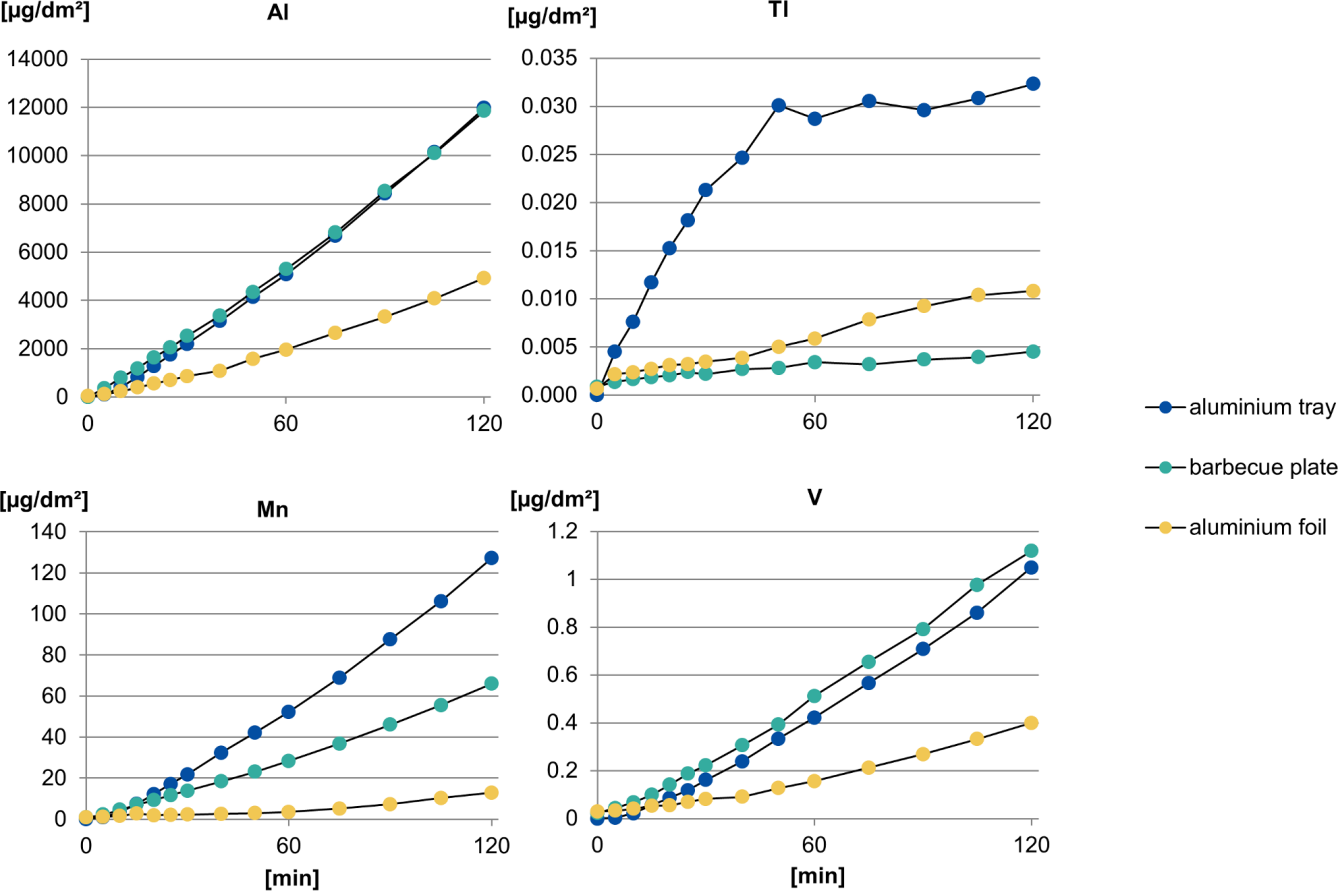
Comparison of the release of Al, Tl, Mn and V between menu tray, grill tray and aluminium foil.

The release of further components like vanadium (V) and manganese (Mn) from the menu trays, a grill tray and aluminium foil has also been analysed. In contrast to thallium, these elements showed a behaviour comparable to aluminium (Fig 6).

Fig 6 also demonstrates that all samples showed a release of thallium. Compared to the grill tray and the foil, the slope in release of thallium from the tray was significantly greater and was followed by stagnation. The source of thallium is not known but experiments in material science have been described which showed thallium inclusions by ion implementation in the outer 150 nm in pure aluminium, as we determined, too [26, 27]. It is described that the process of annealing up to 452°C forms different crystal structures of thallium so that they remain in the aluminium after heating [27]. Thallium is also reported to be a possible impurity in aluminium [28]. Possible contamination through the rolling process, where excipients like oils and filters are used, should be considered.

### Investigations on Cook & Chill

**Fig 7:**
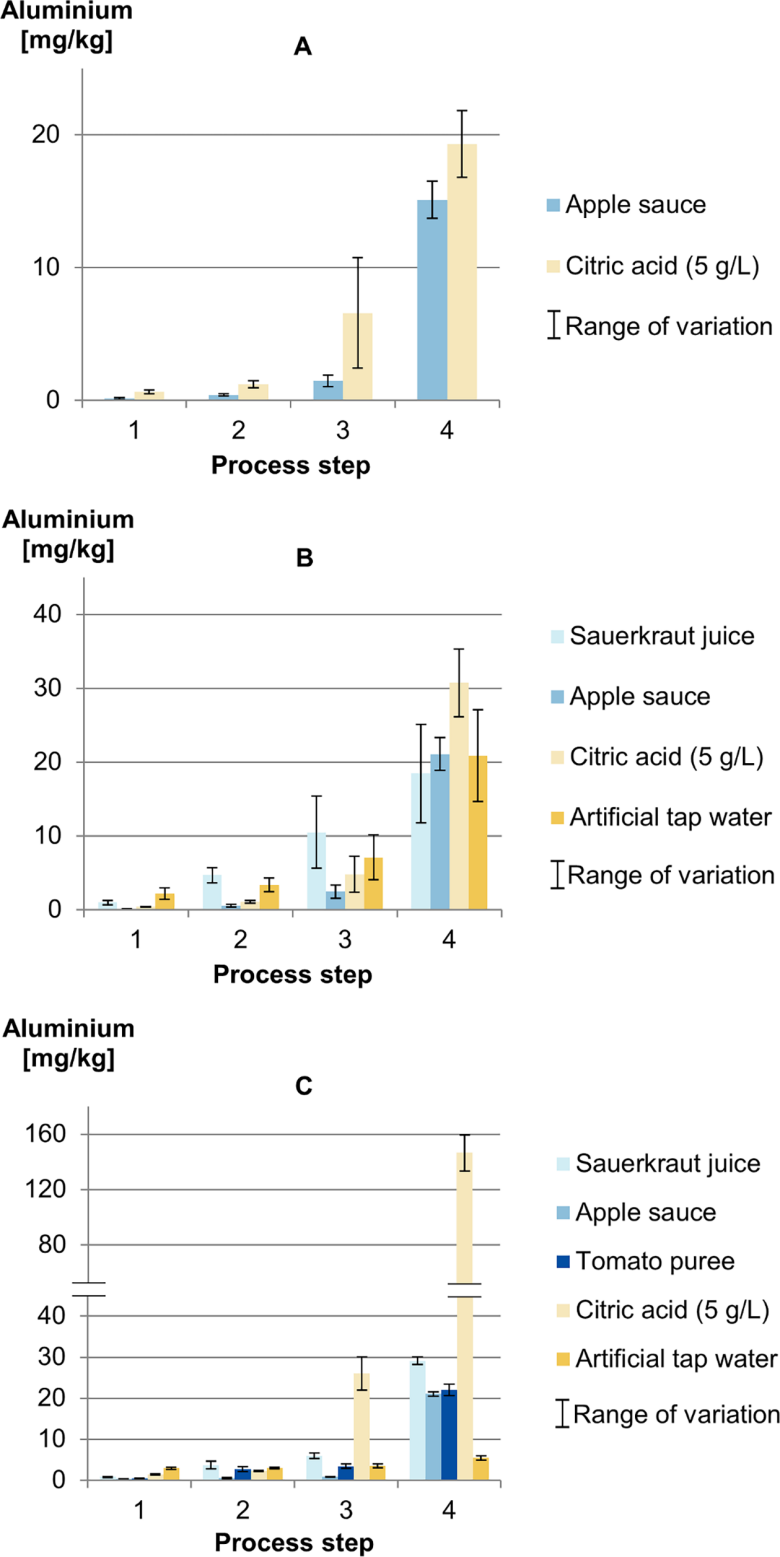
Release of aluminium into benchmark foods and food simulants under Cook & Chill-conditions depending on the process step (A) one-compartment tray, (B) two-compartment trays, (C) three-compartment tray.

In Fig 7, the aluminium releases are given as indicated for the process steps. Hot filling and cooling didn’t cause a dramatic release of aluminium into the foodstuff or simulant with all trays. After storage at 3°C for three days, sauerkraut juice and ATW showed slightly increased releases of aluminium by nearly reaching the SRL of 5 mg/kg for the two- and three-compartment trays. Heating up to 72°C core temperature of the food for two minutes caused significant releases of aluminium up to 5 mg/kg for tomato puree, 10 mg/kg for sauerkraut juice and up to 30 mg/L for 5 g/L citric acid solution. In apple sauce, a maximum of 2.5 mg/kg was reached in the two-compartment trays. The contact of foodstuffs and simulants with the lid was also observed, but this could not be quantified and taken into consideration in the calculation of the results.

After storage for two hours, all foodstuffs reached the SRL for aluminium or exceeded it by up to six times. The leaching of aluminium caused under acidic conditions and high temperatures is also well known for aluminium foil with releases of up to 16 mg Al/kg or 100 μg/cm^2^ [29–31]. Our findings are comparable with values reported by Rajwanshi where cooking tomatoes for 10–30 minutes resulted in a range of 3.1–46.4 mg Al/L [32]. In her review, citric acid (pH 3.0) was shown to be suitable as a food simulant with releases of 4.5–63.0 mg Al/L. In our study 5 g/L citric acid (pH 2.4) was also comparable to the benchmark foodstuffs for the one- and two-compartment tray. It shows up to the sixfold for the three-compartment tray. When used as a food simulant, it is required that citric acid (5 g/L) exceeds the release into foodstuffs, but it was also shown that this can be up to six times the amount, which may be a little too strict.

**Fig 8:**
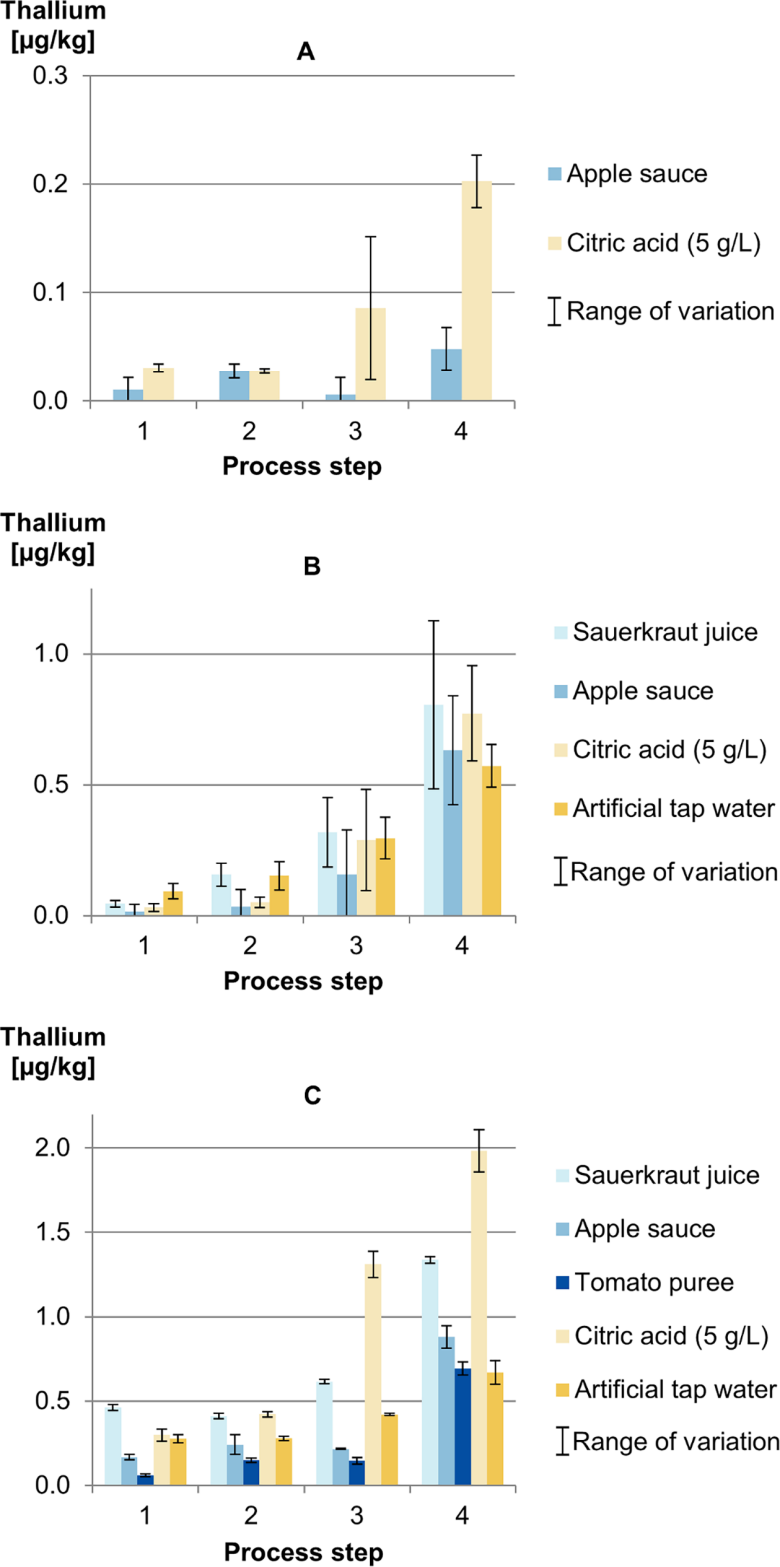
Release of thallium into benchmark foods and food simulants under Cook & Chill-conditions depending on the process step (A) one-compartment tray, (B) two-compartment trays and (C) a three-compartment tray.

Fig 8 shows the results of the thallium releases in the Cook & Chill experiment. As determined in the kinetic experiments, the release of thallium seems to follow another rule than aluminium does. In all experiments, significant levels of thallium were detected after the first process step in contrast to the releases of Al. In the three-compartment tray in particular, thallium is present in a relatively high concentration of 0.1 to 0.5 μg/kg after the first process step and remains constant until the fourth, although the concentration of aluminium increases slightly through the second and third process steps. The concentration of aluminium also increases up to sixfold in the last process step when the concentration of thallium doubles at the most. This is another indication that thallium occurs in the outer areas of the material.

### Investigation on camping pots

**Fig 9:**
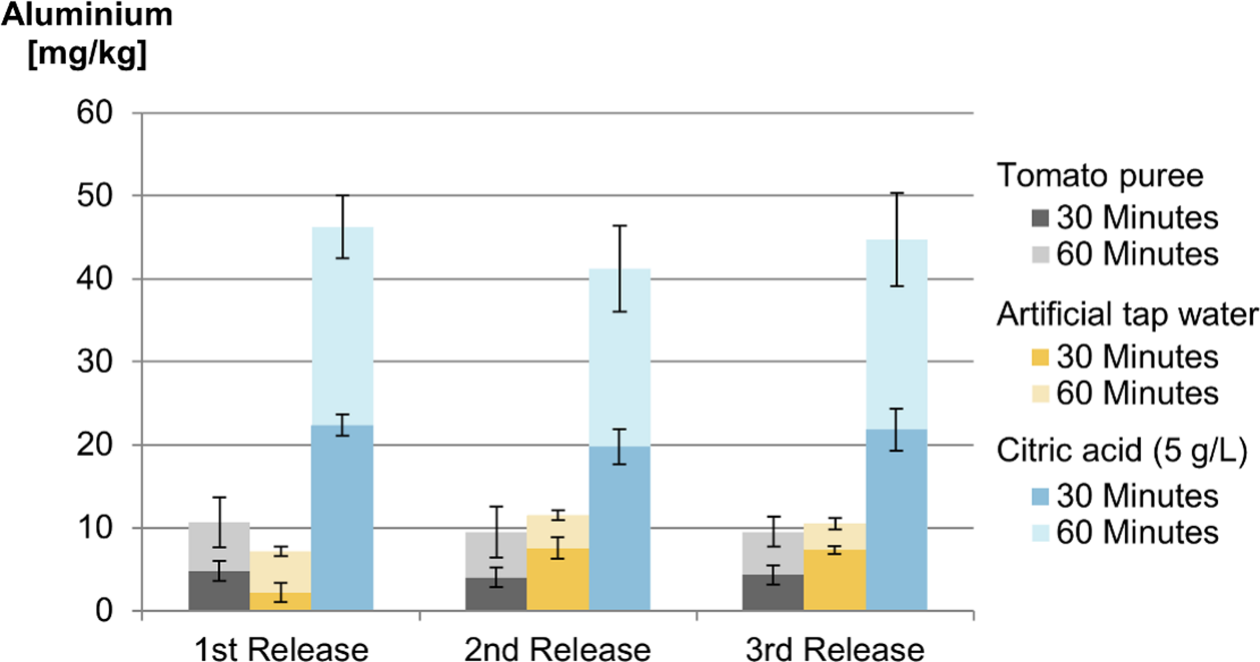
**Release of aluminium into tomato puree, artificial tap water and 0.5% citric acid solution of camping pots at boiling point (n = 3)**.

Camping cookware was tested with tomato puree, 5 g/L citric acid solution and ATW at boiling point. Three subsequent release tests were carried out. Samples were taken after half an hour and one hour. Concentrations of aluminium in tomato puree showed no significant differences between the first, second and third release (Fig 9). With 45 mg aluminium per kg on average, the release in 5 g/L citric acid solution was around four times higher than that in tomato puree (on average 10 mg aluminium per kg). For both testing media, the release doubled after doubling the time. These results correspond with the findings of the investigations with aluminium trays. Again, this concurs with the earlier notion that a low pH is the most influential factor in aluminium release beside the temperature and the contact time. In the case of ATW, the first release test led to significantly lower release levels compared to the second and third. This corresponds to the findings from the kinetic experiments, where the initial dissolution of the oxide layer reduced the release of aluminium. In those kinetic experiments, the influencing factor was the temperature, whereas here we noticed an influence of the pH of the food/simulant. But again after the dissolution of the oxide layer, a constant release behaviour can be seen.

The release of thallium in camping pots was not detectable in tomato puree. In ATW and 5 g/L citric acid solution, thallium was detectable only at very low concentrations of 0.001–0.003 μg/kg.

## Conclusion

In this study, the processes of aluminium release into the acid food simulant citric acid could be attributed to a slow initial dissolution of the outer oxide layer and a subsequent quicker and uniform dissolution of the pure metal. From the latter process, the *E*_*A*_ was derived to be 62 kJ/mol. In addition, a novel quick method for the determination of the *E*_*A*_ using cooling down experiments was developed. With this method, it was possible to extend the temperature range for the calculation of *E*_*A*_ to lower temperatures, showing the validity of the derived value over a broad temperature range. The *E*_*A*_ of 68 kJ/mol derived in this approach matches up well with the first experiments at different constant temperatures. The findings were also confirmed by a recalculation of literature data. Considering the lag time, coherent results were achieved in the calculations of the *E*_*A*_.

For the first time, thallium was found to be released in substantial amounts from food contact materials made of aluminium. It could be demonstrated that the thallium originates mainly from the surface of the aluminium articles, not from the bulk. This might be due to an impurity in the bulk aluminium which is transferred onto the surface during the mechanical treatment of the foil or to a contamination by the rolling oil. The source of this contamination should be elucidated.

It could be demonstrated that the use of uncoated aluminium trays in the Cook & Chill process results in releases of aluminium which can reach the SRL of 5 mg/kg set by the Council of Europe. If subsequently stored at elevated temperatures, as is the case with mobile catering services, high amounts of aluminium can be detected in the acidic benchmark foodstuffs and food simulants.

Given the high overall aluminium exposure of the general population efforts should be made to minimise additional exposure wherever possible. This holds particularly true for avoidable sources such as aluminium food trays used within the Cook & Chill process. The latter are of special significance since they are routinely used in catering settings of nurseries and care homes, thus unnecessarily increasing aluminium intake of vulnerable consumer groups such children and elderly people [33].

Both, the aluminium tray and the camping cookware for repeated use show high releases of aluminium. The frequent use of such kitchenware for the preparation of acidic food should be avoided.

## Supporting information

S1 Table Volumes and surface area of aluminium food trays and camping saucepan. (TIF)

S2 Table Chemicals and elements. (TIF)

S3 Table Instrumental setup ICP-MS (Thermo; icapQ with Prepfast-autosampler). (TIF)

S4 Table Validation data with LOD, LOQ and recoveries taken at concentrations of 0.08, 0.8 and 8 times of the SRL (*n = 7, **n = 6, ***n = 5). (TIF)

S1 Fig Temperature process in one-parted tray (A1) of cooling down (A) and reheating (B).
(TIF)

S2 Fig Temperature process in two-parted trays (A2, A3) of cooling down (A, B) and reheating (C, D).
(TIF)

S3 Fig Temperature process in three-parted tray (A4) of cooling down (A, B) and reheating (C).
(TIF)
