# Supplementary figures and images for: Release of aluminium and thallium ions from uncoated food contact materials made of aluminium alloys into food and food simulant
Source: PLoS One. 2018 Jul 23;13(7):e0200778. doi: 10.1371/journal.pone.0200778 (PMC6056035; doi:10.1371/journal.pone.0200778)

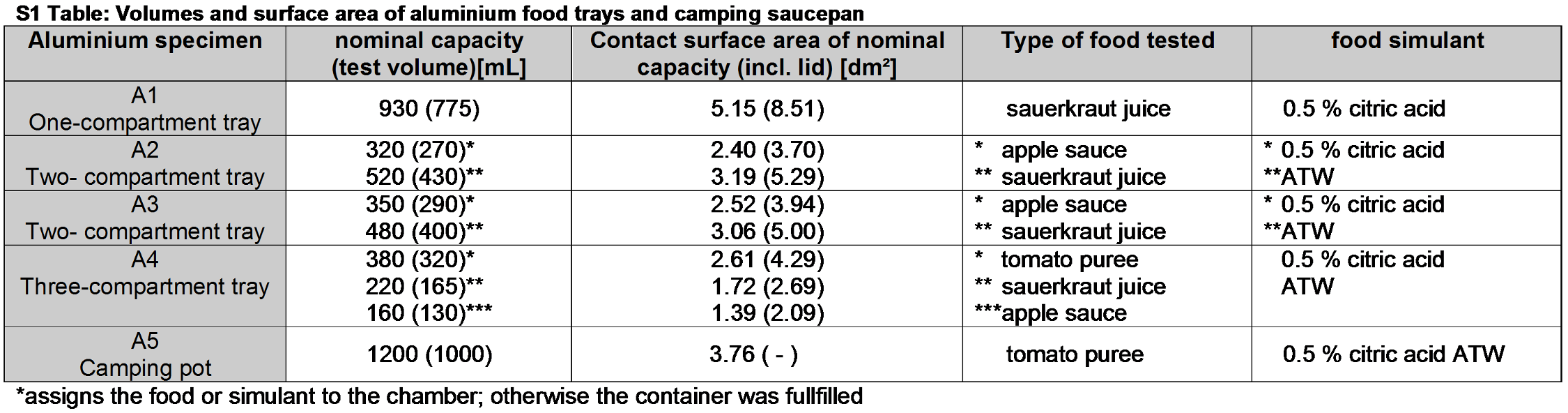

Supplement: S1 Table — (TIF) [file pone.0200778.s001.tif]

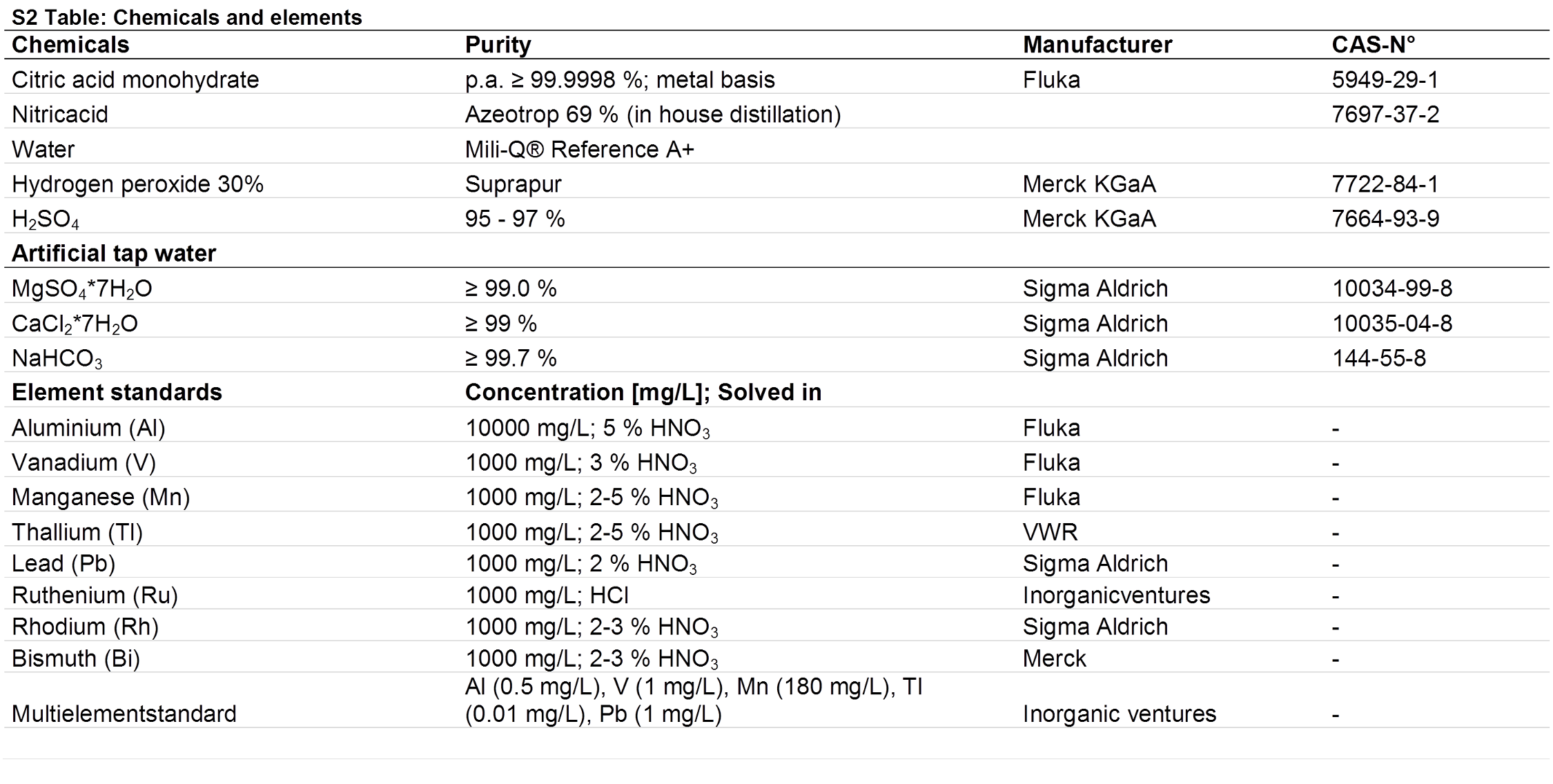

Supplement: S2 Table — (TIF) [file pone.0200778.s002.tif]

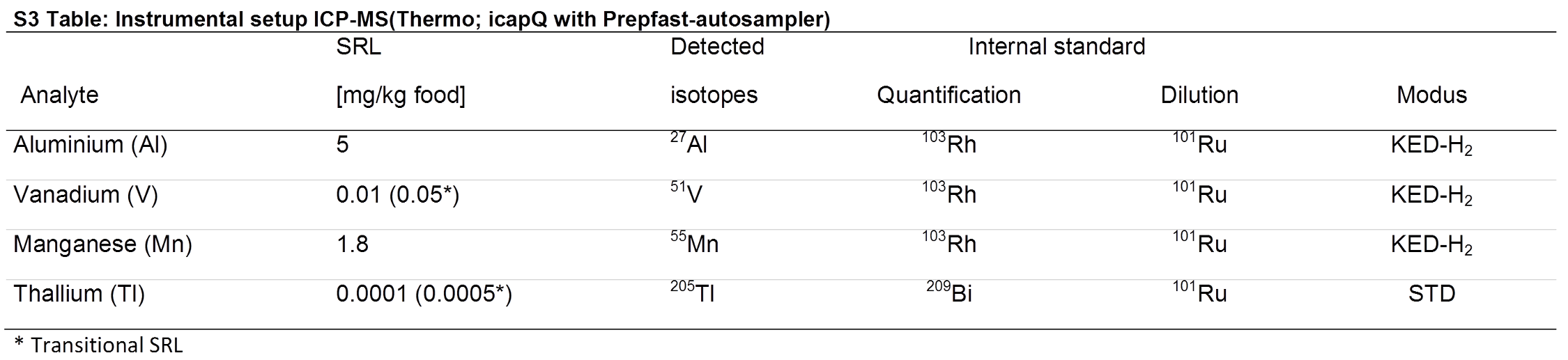

Supplement: S3 Table — (TIF) [file pone.0200778.s003.tif]

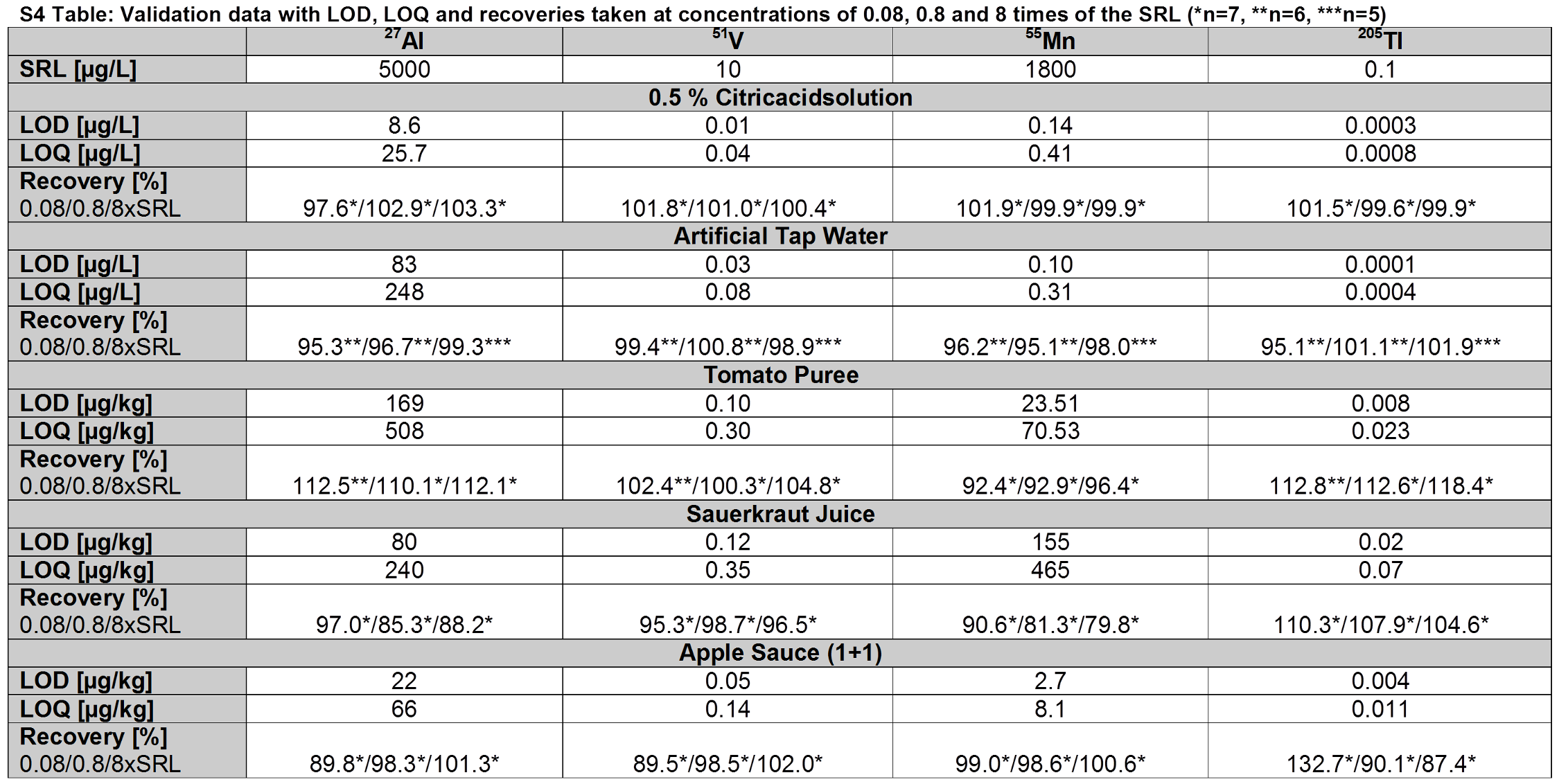

Supplement: S4 Table — (TIF) [file pone.0200778.s004.tif]

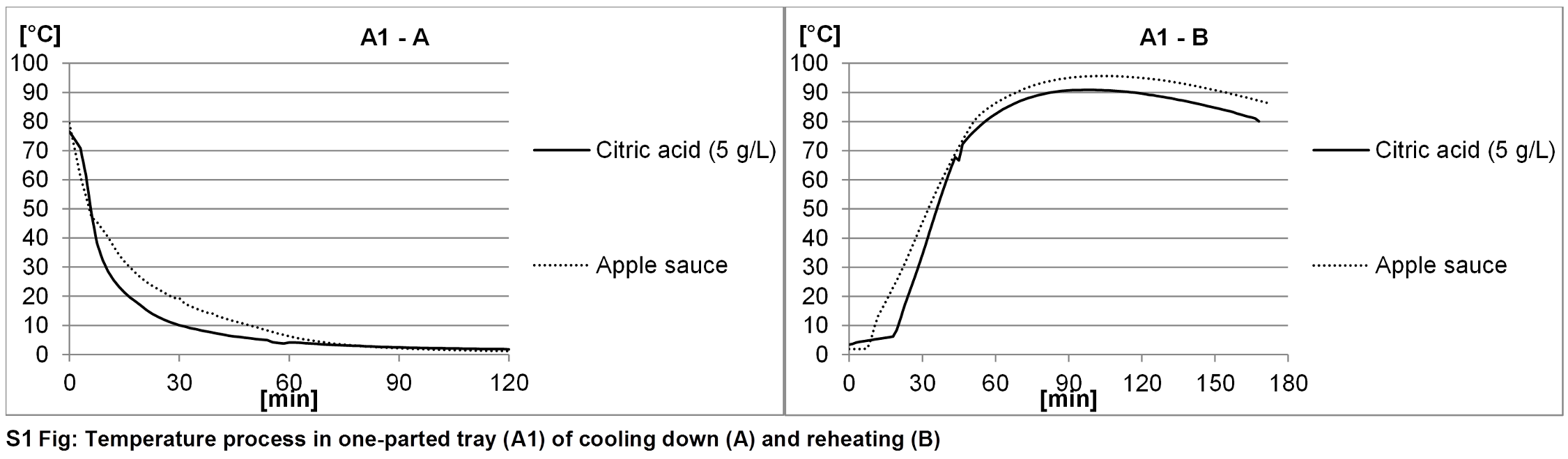

Supplement: S1 Fig — Temperature process in one-parted tray (A1) of cooling down (A) and reheating (B). (TIF) [file pone.0200778.s005.tif]

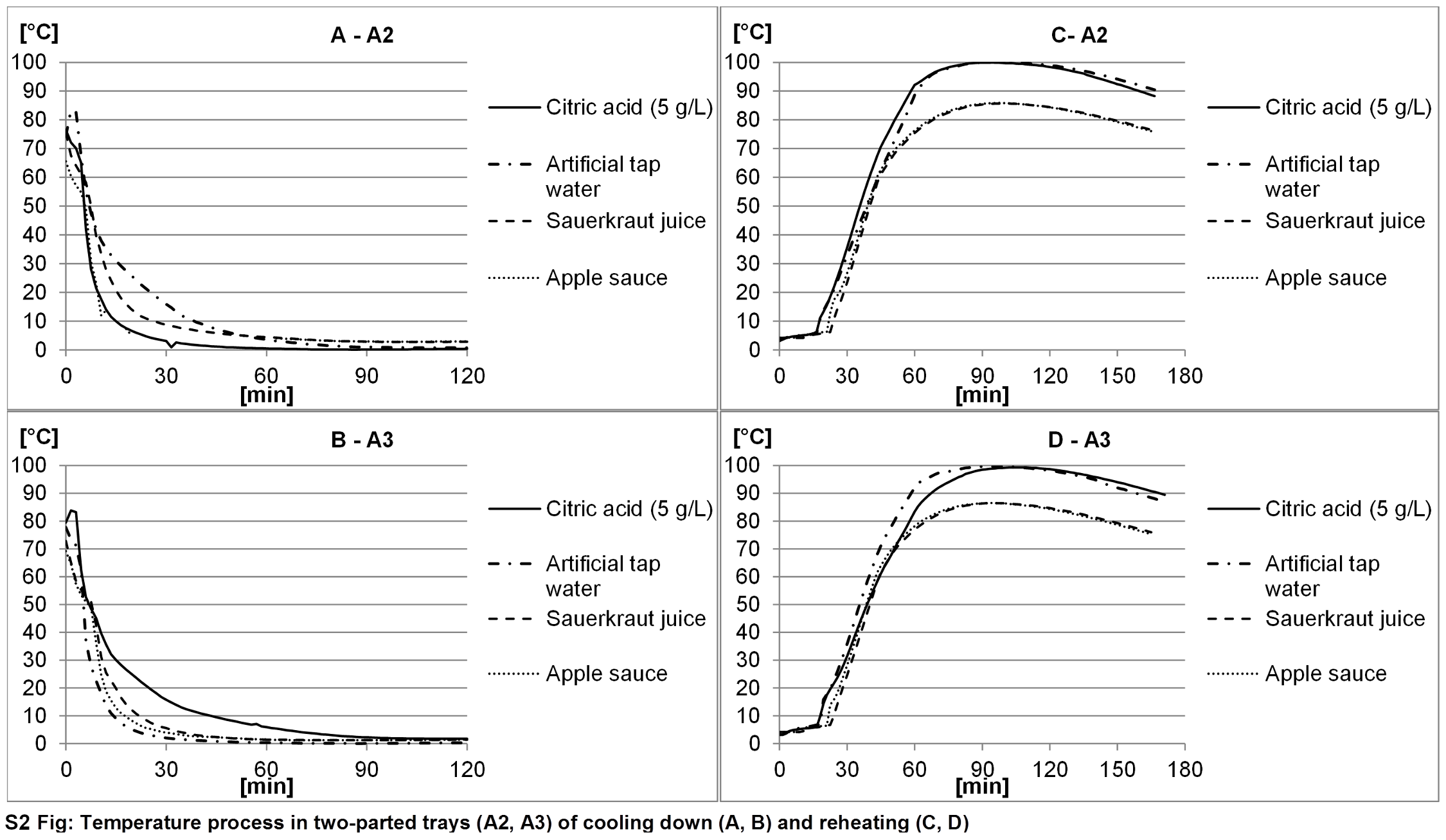

Supplement: S2 Fig — Temperature process in two-parted trays (A2, A3) of cooling down (A, B) and reheating (C, D). (TIF) [file pone.0200778.s006.tif]

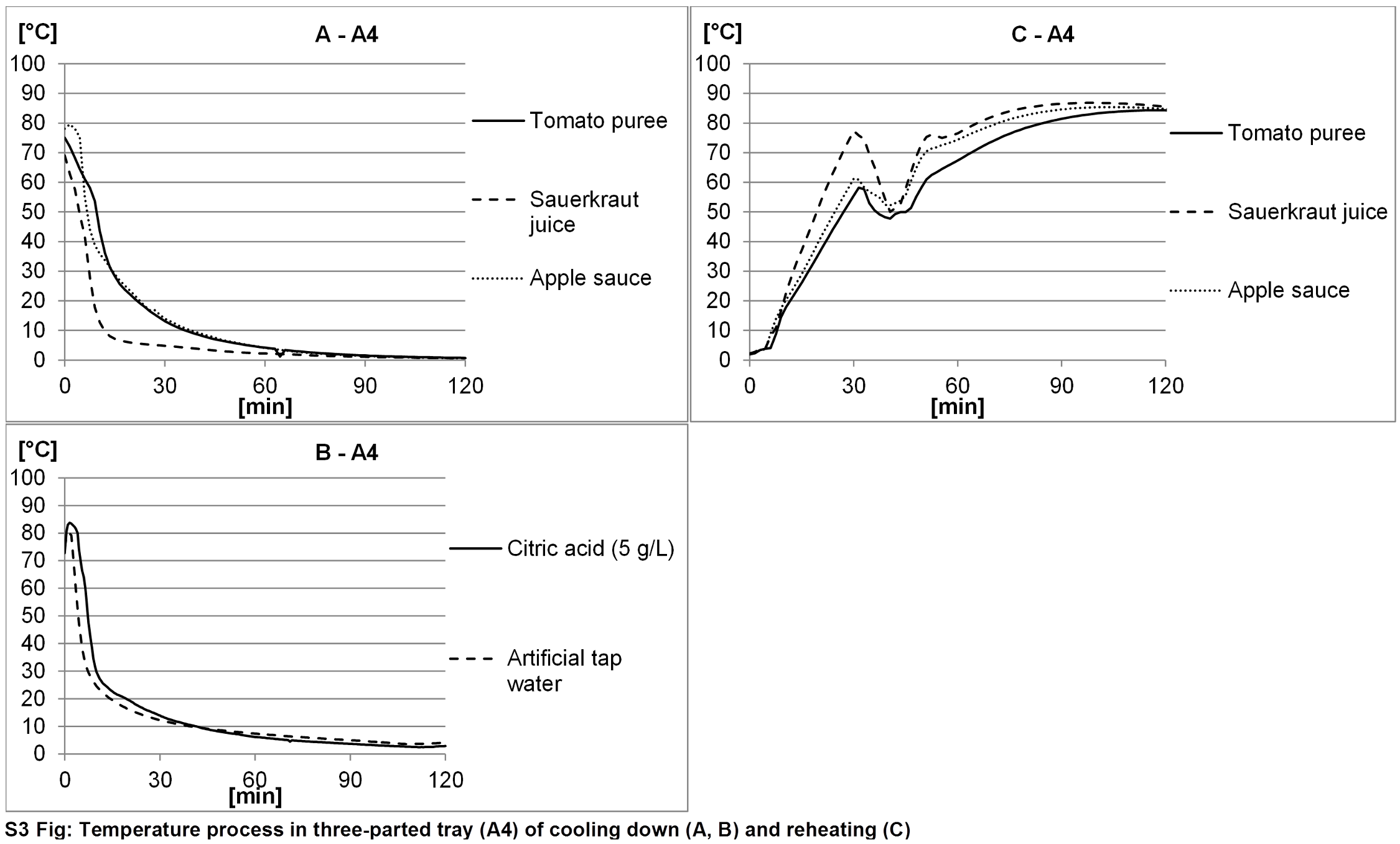

Supplement: S3 Fig — Temperature process in three-parted tray (A4) of cooling down (A, B) and reheating (C). (TIF) [file pone.0200778.s007.tif]
